# Joint Estimation of DOA and Frequency of Multiple Sources with Orthogonal Coprime Arrays

**DOI:** 10.3390/s19020335

**Published:** 2019-01-15

**Authors:** Kai-Chieh Hsu, Jean-Fu Kiang

**Affiliations:** Department of Electrical Engineering, National Taiwan University, Taipei 106, Taiwan; b03901026@ntu.edu.tw

**Keywords:** array signal processing, direction-of-arrival, carrier frequency, coprime array

## Abstract

A two-stage method is proposed to jointly estimate the direction-of-arrival (DOA) and carrier frequency (CF) of multiple sources, by using two orthogonal coprime arrays (CPAs). The DOAs of CF-known sources are estimated first by applying a spatial smoothing MUSIC algorithm. The contribution of these source signals is then removed from the originally received signal by applying an orthogonal complement projector. Next, a joint-ESPRIT algorithm is applied to estimate the DOAs and CFs of the remaining CF-unknown sources. With two orthogonal CPA(5, 6), the RMSE of DOA and CF of applying the proposed method to 30 sources, 13 of which have unknown CF, is less than 1% at SNR >5 dB.

## 1. Introduction

The direction-of-arrival (DOA) of source signals is a prerequisite in many applications like cognitive radar and antenna beamforming. Typical algorithms for estimating the DOAs include subspace-based MUSIC [1], ESPRIT [2] and sparsity-based compressive sensing (CS) approach [3], among others. However, these algorithms are applicable only when the carrier frequency (CF) of signal sources is known [1,2,3,4]. For subspace-based algorithms, MUSIC [1] can generate multiple steering vectors and calculate their projection norm in the null space of receiving signals. On the other hand, ESPRIT [2] can calculate the phase delays if the sensor array possesses rotational invariance, then the DOA can be estimated under a known carrier frequency. As for compressed sensing-based algorithms [4], minimization of $\ell_{1}$-norm is imposed on the sensing matrix to determine the sparse coding. However, due to Doppler shift [5] or in applications of radar tracking [6] and cognitive radios [7,8], the carrier frequency of signal sources can be ambiguous.

Recently, several algorithms were proposed to jointly estimate the DOA and CF of source signals [7,8,9,10]. In [9], a joint angle-frequency estimation (JAFE) algorithm was proposed, in which both the CF and DOA were iteratively updated from their previous values. In [7,8], a compressed carrier and DOA estimation (CaSCADE) algorithm was proposed. A modulated wideband converter (MWC) was used to estimate the CF at sub-Nyquist rate. Then, a CS approach, a parallel factor (PARAFAC) approach and a joint-ESPRIT (JE) were applied to jointly estimate the CF and DOA. In [10], a two-stage method on an uniform linear array (ULA) was proposed. The frequency components of the received signal were determined by applying Fourier transform and spectrum peak searching. Candidate DOAs at each frequency component were estimated with subspace-based methods like MUSIC. After all the CFs and DOAs were estimated, an exhaustive matching algorithm was applied to match each CF to one DOA, at high computational cost. In practice, the received signal is contributed by a combination of CF-known sources and CF-unknown sources. The performance of estimation algorithms like JE degrade as the number of signal sources increases [7,8].

A Khatri–Rao (KR) subspace was proposed to extend the degrees of freedom (DOFs) of an *N*-element ULA from N−1 to 2N−2 so that DOAs of more signal sources can be estimated [11]. In [12,13], KR subspace-based algorithms were proposed to reduce the uncertainty in gain and phase of sensors. Different array configurations such as nested array [14], coprime array (CPA) [15,16] were proposed to further increase the DOFs. In [4], two types of generalized CPA structure were reviewed and their DOFs were estimated. In [17], the DOF of a CPA was extended by scavenging the fourth-order statistics of the received signals. In [18], a triply primed array (TPA), based on fourth-order statistics, was proposed to increase the DOF beyond CPAs. The effects of mutual interference between sensors in an array were also discussed in [19,20,21].

In applications of beamforming [15,16] or cognitive radios [22], a large number of source signals are usually involved. Previous algorithms for joint estimation of DOA and CF [7,8,9,10] were applicable to a limited number of sources. In this work, a two-stage algorithm is proposed to estimate the DOAs of signal sources with known CF first, followed by the JE algorithm to estimate the DOAs and CFs of the remaining signal sources. Two orthogonal CPAs are used to increase the DOFs. This paper is organized as follows. The signal model is presented in Section 2, the proposed two-stage joint estimation algorithm is presented in Section 3, the simulation results are discussed in Section 4 and, finally, some conclusions are drawn in Section 5.

## 2. Signal Model

### 2.1. Sub-Nyquist Sampling Scheme

**Figure 1:**
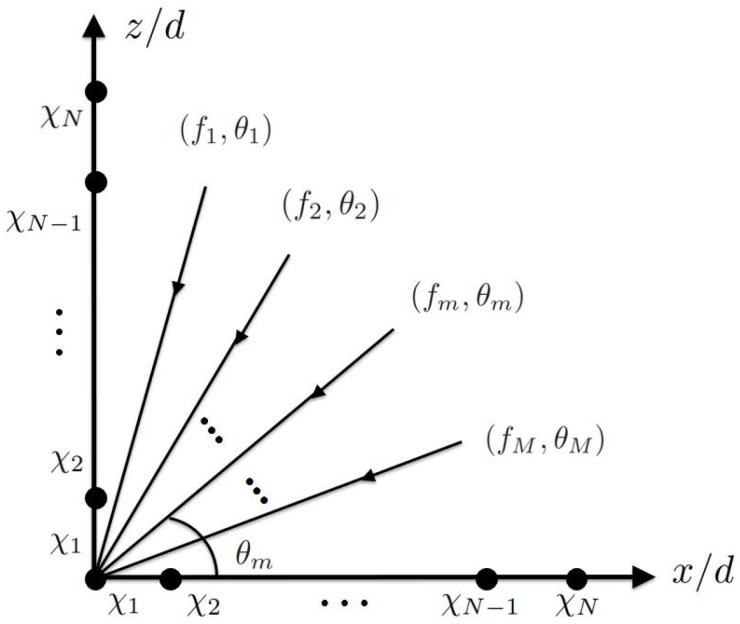
Orthogonal CPAs deployed along *x* and *z* axes, respectively, to estimate DOAs of *M* sources.

In this work, a sub-Nyquist sampling scheme [7,8] is used, where the sampling frequency is on the same order as the bandwidth of source signals, which is much lower than the carrier frequency. Figure 1 shows two orthogonal CPAs deployed along *x* and *z* axes, respectively, to receive signals from *M* sources, with the CF and DOA of the *m*-th source ($s_{m}\left(t\right)$) being $f_{m}$ and $\theta_{m}$, respectively. Without loss of generality, assume that all the signals propagate in the xz plane and are uncorrelated to one another. Also assume the first $M_{c}$ sources have the same CF of $f_{0}$. The bandwidth of each source signal is limited to B=1/T, where *T* is the period of baseband signals. The other signals with unknown CF are assumed to fall in disjoint bands, namely, $\min_{m,n>M_{c},\;m\neq n}\left|f_{m}-f_{n}\right|>B$.

**Figure 2:**
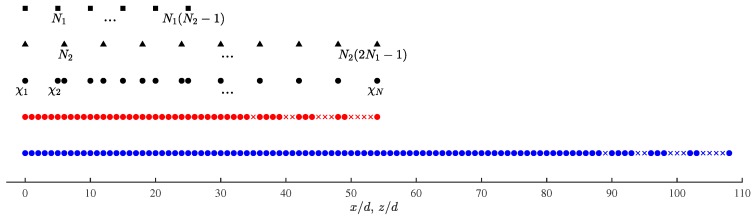
Configuration of CPA($N_{1}$, $N_{2}$), $N_{1}=5$, $N_{2}=6$, ■: first ULA at spacing $N_{1}d$, ▲: second ULA at spacing $N_{2}d$, •: physical array of CPA ($N_{1}$, $N_{2}$), •: second-order virtual array, ×: second-order holes, •: fourth-order virtual array, ×: fourth-order holes.

Figure 2 shows the configuration of a CPA ($N_{1}$, $N_{2}$), which is composed of two ULAs at spacings of $N_{1}d$ and $N_{2}d$, respectively; where $N_{1}$ and $N_{2}$ are coprime integers and *d* is the unit spacing. The first ULA has $N_{2}$ sensors at spacing of $N_{1}d$ and the second ULA has $2N_{1}$ sensors at spacing of $N_{2}d$. Both ULAs share the same first sensor, which is located at the origin, leading to a total of $N=2N_{1}+N_{2}-1$ physical sensors in the CPA ($N_{1}$, $N_{2}$) configuration. The locations of the physical sensors in these two ULAs and the whole CPA are specified by $\Gamma_{1}$, $\Gamma_{2}$ and $\Gamma_{p}$, respectively, with

$$\begin{array}{c} \Gamma_{1}=\left\{n_{2}N_{1}d\;|\;0\leq n_{2}\leq N_{2}-1\right\}\\ \Gamma_{2}=\left\{n_{1}N_{2}d\;|\;0\leq n_{1}\leq 2N_{1}-1\right\}\\ \Gamma_{p}=\Gamma_{1}\cup \Gamma_{2}=\left\{\chi_{1}d,\chi_{2}d,\cdots ,\chi_{N}d\right\} \end{array}$$

where $\chi_{1}<\chi_{2}<\cdots <\chi_{N}$.

The received signal by the *n*-th sensor along the *x* axis can be represented as

$$\begin{array}{c} u_{n}\left(t\right)=\sum_{m=1}^{M}s_{m}\left(t+\chi_{n}\tau_{x}\left(\theta_{m}\right)\right)e^{j2\pi f_{m}\left(t+\chi_{n}\tau_{x}\left(\theta_{m}\right)\right)}≃\sum_{m=1}^{M}s_{m}\left(t\right)e^{j2\pi f_{m}\left(t+\chi_{n}\tau_{x}\left(\theta_{m}\right)\right)} \end{array}$$

where $\tau_{x}\left(\theta \right)=d\cos\theta /c$, and $\chi_{n}\tau_{x}\left(\theta \right)$ is the time advance of the *n*-th sensor with respect to the first one at the origin. The Fourier transform of $u_{n}\left(t\right)$ is

$$\begin{array}{c} U_{n}\left(f\right)=\int_{-\infty }^{\infty }dtu_{n}\left(t\right)e^{-j2\pi ft}=\sum_{m=1}^{M}e^{j2\pi f_{m}\chi_{n}\tau_{x}\left(\theta_{m}\right)}\int_{-\infty }^{\infty }dts_{m}\left(t\right)e^{-j2\pi (f-f_{m})t}\\ =\sum_{m=1}^{M}S_{m}\left(f-f_{m}\right)e^{j2\pi f_{m}\chi_{n}\tau_{x}\left(\theta_{m}\right)} \end{array}$$

where $S_{m}\left(f\right)$ is the spectrum of the *m*-th baseband signal, with |f|≤B/2.

Consider a periodic signal $p\left(t\right)=\sum_{v=-\infty }^{\infty }c_{v}e^{j2\pi f_{p}vt}$ with period $T_{p}=1/f_{p}$, where $c_{v}=\frac{1}{T_{p}}\int_{0}^{T_{p}}p\left(t\right)e^{-j2\pi f_{p}vt}dt$. The received signal $u_{n}\left(t\right)$ is multiplied with p(t) to derive

$$\begin{array}{c} {\tilde{x}}_{n}\left(t\right)=p\left(t\right)u_{n}\left(t\right)=\sum_{m=1}^{M}e^{j2\pi f_{m}\chi_{n}\tau_{x}\left(\theta_{m}\right)}\sum_{v=-\infty }^{\infty }c_{v}s_{m}\left(t\right)e^{j2\pi (f_{m}+vf_{p})t} \end{array}$$

which is Fourier transformed to have

$$\begin{array}{c} \;{\tilde{X}}_{n}\left(f\right)=\int_{-\infty }^{\infty }dt{\tilde{x}}_{n}\left(t\right)e^{-j2\pi ft}=\sum_{m=1}^{M}e^{j2\pi f_{m}\chi_{n}\tau_{x}\left(\theta_{m}\right)}\sum_{v=-\infty }^{\infty }c_{v}S_{m}\left(f-f_{m}-vf_{p}\right) \end{array}$$

By applying an ideal low-pass filter (LPF) with cutoff frequency $f_{c}/2$ to ${\tilde{X}}_{n}\left(f\right)$, we obtain

$$\begin{array}{c} X_{n}\left(f\right)=\left\{\begin{array}{cc} {\tilde{X}}_{n}\left(f\right),\; & \left|f\right|\leq f_{c}/2\\ 0, & \mathrm{otherwise} \end{array}\right. \end{array}$$

By choosing $f_{c}>B$, the baseband information in $S_{m}\left(f\right)$’s is preserved in $X_{n}\left(f\right)$. Explicitly, $X_{n}\left(f\right)$ can be represented as

$$\begin{array}{c} X_{n}\left(f\right)=\sum_{m=1}^{M}{\tilde{S}}_{m}\left(f\right)e^{j2\pi f_{m}\chi_{n}\tau_{x}\left(\theta_{m}\right)} \end{array}$$

with ${\tilde{S}}_{m}\left(f\right)=\sum_{v=-V_{1}}^{V_{1}}c_{v}S_{m}\left(f-f_{m}-vf_{p}\right)$, where $V_{1}=\left\lceil \left(f_{c}+f_{m}\right)/\left(2f_{p}\right)\right\rceil -1$, which is slightly different from that in [8].

Next, take the inverse Fourier transform of $X_{n}\left(f\right)$ to have

$$\begin{array}{c} x_{n}\left(t\right)=\sum_{m=1}^{M}e^{j2\pi f_{m}\chi_{n}\tau_{x}\left(\theta_{m}\right)}{\tilde{s}}_{m}\left(t\right) \end{array}$$

which is sampled at the rate of $f_{s}=1/T_{s}$ to obtain

(1) $$\begin{array}{c} x_{n}\left[\ell \right]=\sum_{m=1}^{M}e^{j2\pi f_{m}\chi_{n}\tau_{x}\left(\theta_{m}\right)}w_{m}\left[\ell \right] \end{array}$$

where $x_{n}\left[\ell \right]=x_{n}\left(\ell T_{s}\right)$ and $w_{m}\left[\ell \right]={\tilde{s}}_{m}\left(\ell T_{s}\right)$. Note that we may choose $f_{s}=f_{c}$.

Equation (1) can be put in a matrix form as

(2) $$\begin{array}{c} \bar{x}\left[\ell \right]={\bar{\bar{A}}}_{x}\left(\bar{f},\bar{\theta }\right)\cdot \bar{w}\left[\ell \right] \end{array}$$

where $\bar{x}\left[\ell \right]={\left[x_{1}\left[\ell \right],x_{2}\left[\ell \right],\cdots ,x_{N}\left[\ell \right]\right]}^{t}$, $\bar{w}\left[\ell \right]={\left[w_{1}\left[\ell \right],w_{2}\left[\ell \right],\cdots ,w_{M}\left[\ell \right]\right]}^{t}$, $\bar{f}={\left[f_{1},f_{2},\cdots ,f_{M}\right]}^{t}$, $\bar{\theta }={\left[\theta_{1},\theta_{2},\cdots ,\theta_{M}\right]}^{t}$ and

$$\begin{array}{c} {\bar{\bar{A}}}_{x}\left(\bar{f},\bar{\theta }\right)=\left[\begin{array}{ccc} e^{j2\pi f_{1}\chi_{1}\tau_{x}\left(\theta_{1}\right)} & \cdots & e^{j2\pi f_{M}\chi_{1}\tau_{x}\left(\theta_{M}\right)}\\ e^{j2\pi f_{1}\chi_{2}\tau_{x}\left(\theta_{1}\right)} & \cdots & e^{j2\pi f_{M}\chi_{2}\tau_{x}\left(\theta_{M}\right)}\\ \vdots & & \vdots \\ e^{j2\pi f_{1}\chi_{N}\tau_{x}\left(\theta_{1}\right)} & \cdots & e^{j2\pi f_{M}\chi_{N}\tau_{x}\left(\theta_{M}\right)} \end{array}\right] \end{array}$$

Similarly, the received signals in the array along the *z*-axis are processed to have

(3) $$\begin{array}{c} \bar{z}\left[\ell \right]={\bar{\bar{A}}}_{z}\left(\bar{f},\bar{\theta }\right)\cdot \bar{w}\left[\ell \right] \end{array}$$

where $\bar{z}\left[\ell \right]={\left[z_{1}\left[\ell \right],z_{2}\left[\ell \right],\cdots ,z_{N}\left[\ell \right]\right]}^{t}$, $z_{n}\left[\ell \right]=\sum_{m=1}^{M}e^{j2\pi f_{m}\chi_{n}\tau_{z}\left(\theta_{m}\right)}w_{m}\left[\ell \right]$ and

$$\begin{array}{c} {\bar{\bar{A}}}_{z}\left(\bar{f},\bar{\theta }\right)=\left[\begin{array}{ccc} e^{j2\pi f_{1}\chi_{1}\tau_{z}\left(\theta_{1}\right)} & \cdots & e^{j2\pi f_{M}\chi_{1}\tau_{z}\left(\theta_{M}\right)}\\ e^{j2\pi f_{1}\chi_{2}\tau_{z}\left(\theta_{1}\right)} & \cdots & e^{j2\pi f_{M}\chi_{2}\tau_{z}\left(\theta_{M}\right)}\\ \vdots & & \vdots \\ e^{j2\pi f_{1}\chi_{N}\tau_{z}\left(\theta_{1}\right)} & \cdots & e^{j2\pi f_{M}\chi_{N}\tau_{z}\left(\theta_{M}\right)} \end{array}\right] \end{array}$$

with $\tau_{z}\left(\theta \right)=d\sin\theta /c$.

By taking noise into consideration, (2) and (3) are modified as

(4) $$\begin{array}{c} \bar{x}\left[\ell \right]={\bar{\bar{A}}}_{x}\cdot \bar{w}\left[\ell \right]+{\bar{n}}_{x}\left[\ell \right],\;\;\bar{z}\left[\ell \right]={\bar{\bar{A}}}_{z}\cdot \bar{w}\left[\ell \right]+{\bar{n}}_{z}\left[\ell \right] \end{array}$$

where ${\bar{\bar{A}}}_{x}$ and ${\bar{\bar{A}}}_{z}$ are the abbreviations of ${\bar{\bar{A}}}_{x}\left(\bar{f},\bar{\theta }\right)$ and ${\bar{\bar{A}}}_{z}\left(\bar{f},\bar{\theta }\right)$, respectively; ${\bar{n}}_{x}\left[\ell \right]$ and ${\bar{n}}_{z}\left[\ell \right]$ are uncorrelated additive white Gaussian noise vectors at the *ℓ*th sampling time instant. The noise vectors are assumed to have zero mean and covariance matrix $\sigma_{n}^{\prime 2}{\bar{\bar{I}}}_{N}$, where ${\bar{\bar{I}}}_{N}$ is an N×N identity matrix.

### 2.2. Second-Order Statistics

Assume the source signals are wide-sense quasi-stationary, which are observed over *Q* non-overlapped time frames, with each time frame lasting for $LT_{s}$ [4,11,18]. The average power of the *m*-th source signal in the *q*-th time frame is represented as

$$\begin{array}{c} P_{m}^{q}=E\left\{{\left|w_{m}\left[\ell \right]\right|}^{2}\right\},\;\;1\leq q\leq Q,\;\left(q-1\right)L\leq \ell \leq qL-1 \end{array}$$

Assume $P_{m}^{q}$’s from different sources are uncorrelated, with their first and second moments determined as

$$\begin{array}{c} E_{q}\left\{P_{m}^{q}\right\}=\mu_{m},\;\;E_{q}\left\{{\left(P_{m}^{q}-\mu_{m}\right)}^{2}\right\}=\sigma_{m}^{2} \end{array}$$

where $E_{q}$ means the expectation value is taken over the *q*-th time frame. The covariance matrices of the received signals in the *q*-th time frame are determined as

(5) $$\begin{array}{c} {\bar{\bar{R}}}_{x}^{q}=E\left\{{\bar{x}}^{q}\left[\ell \right]{\bar{x}}^{q†}\left[\ell \right]\right\}={\bar{\bar{A}}}_{x}\cdot {\bar{\bar{D}}}^{q}\cdot {\bar{\bar{A}}}_{x}^{†}+\sigma_{n}^{\prime 2}{\bar{\bar{I}}}_{N}\\ {\bar{\bar{R}}}_{z}^{q}=E\left\{{\bar{z}}^{q}\left[\ell \right]{\bar{z}}^{q†}\left[\ell \right]\right\}={\bar{\bar{A}}}_{z}\cdot {\bar{\bar{D}}}^{q}\cdot {\bar{\bar{A}}}_{z}^{†}+\sigma_{n}^{\prime 2}{\bar{\bar{I}}}_{N} \end{array}$$

where ${\bar{x}}^{q}\left[\ell \right]$ is the received signal in the *q*-th time frame and ${\bar{\bar{D}}}^{q}=\mathrm{diag}\left\{P_{1}^{q},P_{2}^{q},\cdots ,P_{M}^{q}\right\}$.

In practice, ${\bar{\bar{R}}}_{x}^{q}$ and ${\bar{\bar{R}}}_{z}^{q}$ are estimated as

(6) $$\begin{array}{c} {\tilde{\bar{\bar{R}}}}_{x}^{q}=\frac{1}{L}\sum_{\ell =(q-1)L}^{qL-1}{\bar{x}}^{q}\left[\ell \right]{\bar{x}}^{q†}\left[\ell \right],\;\;{\tilde{\bar{\bar{R}}}}_{z}^{q}=\frac{1}{L}\sum_{\ell =(q-1)L}^{qL-1}{\bar{z}}^{q}\left[\ell \right]{\bar{z}}^{q†}\left[\ell \right] \end{array}$$

By concatenating all the columns of ${\bar{\bar{R}}}_{x}^{q}$ and ${\bar{\bar{R}}}_{z}^{q}$, respectively, into $N^{2}\times 1$ vectors, (5) is transformed to

$$\begin{array}{c} {\bar{y}}_{x}^{\prime }\left[q\right]=\mathrm{vec}\left\{{\bar{\bar{R}}}_{x}^{q}\right\}={\bar{\bar{B}}}_{x}\cdot {\bar{P}}^{\prime }\left[q\right]+\sigma_{n}^{\prime 2}{\bar{\nu }}_{N}\\ {\bar{y}}_{z}^{\prime }\left[q\right]=\mathrm{vec}\left\{{\bar{\bar{R}}}_{z}^{q}\right\}={\bar{\bar{B}}}_{z}\cdot {\bar{P}}^{\prime }\left[q\right]+\sigma_{n}^{\prime 2}{\bar{\nu }}_{N} \end{array}$$

where ${\bar{P}}^{\prime }\left[q\right]={\left[P_{1}^{q},P_{2}^{q},\cdots ,P_{M}^{q}\right]}^{t}$, ${\bar{\nu }}_{N}=\mathrm{vec}\left\{{\bar{\bar{I}}}_{N}\right\}$, ${\bar{\bar{B}}}_{x}={\bar{\bar{A}}}_{x}^{*}⊙{\bar{\bar{A}}}_{x}$ and ${\bar{\bar{B}}}_{z}={\bar{\bar{A}}}_{z}^{*}⊙{\bar{\bar{A}}}_{z}$ are second-order difference manifold matrices, and ⊙ means column-wise Kronecker product. By subtracting the expectation value over time-frame index *q* from ${\bar{y}}_{x}^{\prime }\left[q\right]$ and ${\bar{y}}_{z}^{\prime }\left[q\right]$, respectively, we have

$$\begin{array}{c} {\bar{y}}_{x}\left[q\right]={\bar{y}}_{x}^{\prime }\left[q\right]-E_{q}\left\{{\bar{y}}_{x}^{\prime }\left[q\right]\right\}={\bar{\bar{B}}}_{x}\cdot \bar{P}\left[q\right]\\ {\bar{y}}_{z}\left[q\right]={\bar{y}}_{z}^{\prime }\left[q\right]-E_{q}\left\{{\bar{y}}_{z}^{\prime }\left[q\right]\right\}={\bar{\bar{B}}}_{z}\cdot \bar{P}\left[q\right] \end{array}$$

where $\bar{P}\left[q\right]$ = ${\left[P_{1}^{q}-\mu_{1},P_{2}^{q}-\mu_{2},\cdots ,P_{M}^{q}-\mu_{M}\right]}^{t}$.

Next, by taking the average of entries in ${\bar{y}}_{x}\left[q\right]$ and ${\bar{y}}_{z}\left[q\right]$, respectively, that have the same lags (of same or opposite sign), we derive the observation vectors as

(7) $$\begin{array}{c} {\bar{y}}_{x}^{\Phi }\left[q\right]={\bar{\bar{B}}}_{x}^{\Phi }\cdot \bar{P}\left[q\right],\;\;{\bar{y}}_{z}^{\Phi }\left[q\right]={\bar{\bar{B}}}_{z}^{\Phi }\cdot \bar{P}\left[q\right] \end{array}$$

where Φ contains the index of entries having lags in $\Gamma_{s}=\left\{-N_{s}d,\cdots ,N_{s}d\right\}$, with $N_{s}=N_{1}N_{2}+N_{1}-1$. In other words, ${\bar{\bar{B}}}_{x}^{\Phi }$ and ${\bar{\bar{B}}}_{z}^{\Phi }$ serve as steering matrices of effective ULAs deployed along *x* and *z* axes, respectively, with virtual sensor locations specified by $\Gamma_{s}$. The set Φ associated with the *x*-direction ULA is the same as that with the *z*-direction ULA because both ULAs have the same physical configuration.

## 3. Joint Estimation of DOA and CF

In this Section, a two-stage algorithm is proposed, in which the DOA of CF-known sources are estimated first, then the DOA and CF of the remaining sources are jointly estimated.

### 3.1. DOA Estimation of Signal Sources with Known CF

The second-order statistics of the received signals can be used to derive *Q*-manifold signals, which are then processed with subspace-based algorithms like ESPRIT or MUSIC to estimate the DOA and CF of the original sources. If the fourth-order statistics of the source signals are available, the fourth-order covariance matrices can be estimated as

$$\begin{array}{c} \;{\tilde{\bar{\bar{R}}}}_{xx}=\frac{1}{Q}\sum_{q=1}^{Q}{\bar{y}}_{x}^{\Phi }\left[q\right]{\bar{y}}_{x}^{\Phi †}\left[q\right]≃E\left\{{\bar{y}}_{x}^{\Phi }\left[q\right]{\bar{y}}_{x}^{\Phi †}\left[q\right]\right\}={\bar{\bar{B}}}_{x}^{\Phi }\cdot \bar{\bar{D}}\cdot {\bar{\bar{B}}}_{x}^{\Phi †}\\ \;{\tilde{\bar{\bar{R}}}}_{zz}=\frac{1}{Q}\sum_{q=1}^{Q}{\bar{y}}_{z}^{\Phi }\left[q\right]{\bar{y}}_{z}^{\Phi †}\left[q\right]≃E\left\{{\bar{y}}_{z}^{\Phi }\left[q\right]{\bar{y}}_{z}^{\Phi †}\left[q\right]\right\}={\bar{\bar{B}}}_{z}^{\Phi }\cdot \bar{\bar{D}}\cdot {\bar{\bar{B}}}_{z}^{\Phi †} \end{array}$$

where $\bar{\bar{D}}=\mathrm{diag}\left\{\sigma_{1}^{2},\sigma_{2}^{2},\cdots ,\sigma_{M}^{2}\right\}$. The covariance matrices ${\tilde{\bar{\bar{R}}}}_{xx}$ and ${\tilde{\bar{\bar{R}}}}_{zz}$ are then vectorized as

(8) $$\begin{array}{c} {\bar{y}}_{xx}=\mathrm{vec}\left\{{\bar{\bar{R}}}_{xx}\right\}={\bar{\bar{C}}}_{x}\cdot \bar{P}\\ {\bar{y}}_{zz}=\mathrm{vec}\left\{{\bar{\bar{R}}}_{zz}\right\}={\bar{\bar{C}}}_{z}\cdot \bar{P} \end{array}$$

where ${\bar{\bar{C}}}_{x}={\bar{\bar{B}}}_{x}^{*}⊙{\bar{\bar{B}}}_{x}$, ${\bar{\bar{C}}}_{z}={\bar{\bar{B}}}_{z}^{*}⊙{\bar{\bar{B}}}_{z}$, and $\bar{P}={\left[\sigma_{1}^{2},\sigma_{2}^{2},\cdots ,\sigma_{M}^{2}\right]}^{t}$. By taking the average of entries with same lags in ${\bar{y}}_{xx}$ and ${\bar{y}}_{zz}$, respectively, we obtain

(9) $$\begin{array}{c} {\bar{y}}_{xx}^{\Phi^{\prime }}={\bar{\bar{C}}}_{x}^{\Phi^{\prime }}\cdot \bar{P},\;\;{\bar{y}}_{zz}^{\Phi^{\prime }}={\bar{\bar{C}}}_{z}^{\Phi^{\prime }}\cdot \bar{P} \end{array}$$

where $\Phi^{\prime }$ contains the index of entries having lags in $\Gamma_{f}=\left\{-N_{f}d,\cdots ,N_{f}d\right\}$, with $N_{f}=3N_{1}N_{2}+N_{1}-N_{2}-1$ [17]. The set $\Phi^{\prime }$ associated with the ULA in *x*-direction is the same as that with the ULA in *z*-direction because both ULAs have the same physical configuration. Note that subspace-based algorithms are applicable to CPA with contiguous lags in both *x* and *z* directions. Next, construct spatial smoothing covariance matrices of the fourth-order as [23]

$$\begin{array}{c} {\bar{\bar{R}}}_{xx}^{\prime }=\left[\begin{array}{cccc} {\bar{y}}_{xx}^{\Phi^{\prime }}\left[1\right] & {\bar{y}}_{xx}^{\Phi^{\prime }}\left[2\right] & \cdots & {\bar{y}}_{xx}^{\Phi^{\prime }}\left[N_{f}+1\right]\\ {\bar{y}}_{xx}^{\Phi^{\prime }}\left[2\right] & {\bar{y}}_{xx}^{\Phi^{\prime }}\left[3\right] & \cdots & {\bar{y}}_{xx}^{\Phi^{\prime }}\left[N_{f}+2\right]\\ \vdots & \vdots & \ddots & \vdots \\ {\bar{y}}_{xx}^{\Phi^{\prime }}\left[N_{f}+1\right] & {\bar{y}}_{xx}^{\Phi^{\prime }}\left[N_{f}+2\right] & \cdots & {\bar{y}}_{xx}^{\Phi^{\prime }}\left[2N_{f}+1\right] \end{array}\right] \end{array}$$

(10) $$\begin{array}{c} {\bar{\bar{R}}}_{zz}^{\prime }=\left[\begin{array}{cccc} {\bar{y}}_{zz}^{\Phi^{\prime }}\left[1\right] & {\bar{y}}_{zz}^{\Phi^{\prime }}\left[2\right] & \cdots & {\bar{y}}_{zz}^{\Phi^{\prime }}\left[N_{f}+1\right]\\ {\bar{y}}_{zz}^{\Phi^{\prime }}\left[2\right] & {\bar{y}}_{zz}^{\Phi^{\prime }}\left[3\right] & \cdots & {\bar{y}}_{zz}^{\Phi^{\prime }}\left[N_{f}+2\right]\\ \vdots & \vdots & \ddots & \vdots \\ {\bar{y}}_{zz}^{\Phi^{\prime }}\left[N_{f}+1\right] & {\bar{y}}_{zz}^{\Phi^{\prime }}\left[N_{f}+2\right] & \cdots & {\bar{y}}_{zz}^{\Phi^{\prime }}\left[2N_{f}+1\right] \end{array}\right] \end{array}$$

Algorithm 1 lists the major steps of the spatial smoothing MUSIC (SS-MUSIC). In steps 5–7, the conventional MUSIC algorithm is applied to ${\bar{\bar{R}}}_{xx}^{\prime }$ and ${\bar{\bar{R}}}_{zz}^{\prime }$, respectively, to estimate the DOAs to the nearest grid points, and record them in ${\bar{\phi }}_{1x}$ and ${\bar{\phi }}_{1z}$, respectively. Next, for every entry in ${\bar{\phi }}_{1z}$, find the best match in ${\bar{\phi }}_{1x}$ with DOA difference smaller than 0.5°, then record the average of these two DOAs in ${\bar{\theta }}_{1}^{\prime }$.

**Algorithm 1** SS-MUSIC based on two orthogonal CPAs
**Input:**
*Q* snapshots of manifold vectors, ${\bar{y}}_{x}^{\Phi }$ and ${\bar{y}}_{z}^{\Phi }$ in (7)Total number of signal sources, *M*Known carrier frequency, $f_{0}$
**Output:**
Estimated DOA vector, ${\bar{\theta }}_{1}^{\prime }$
**Algorithm:**
Estimate fourth-order covariance matrices as
${\tilde{\bar{\bar{R}}}}_{xx}=\frac{1}{Q}\sum_{q=1}^{Q}{\bar{y}}_{x}^{\Phi }\left[q\right]{\bar{y}}_{x}^{\Phi †}\left[q\right],\;$ ${\tilde{\bar{\bar{R}}}}_{zz}=\frac{1}{Q}\sum_{q=1}^{Q}{\bar{y}}_{z}^{\Phi }\left[q\right]{\bar{y}}_{z}^{\Phi †}\left[q\right]$
Vectorize, average and remove non-contiguous lags to form ${\bar{y}}_{xx}^{\Phi^{\prime }}$ and ${\bar{y}}_{zz}^{\Phi^{\prime }}$, as in (8) and (9)Construct spatial smoothing covariance matrices ${\bar{\bar{R}}}_{xx}^{\prime }$ and ${\bar{\bar{R}}}_{zz}^{\prime }$, as in (10)Apply singular-value decomposition (SVD) on ${\bar{\bar{R}}}_{xx}^{\prime }$ and ${\bar{\bar{R}}}_{zz}^{\prime }$ to have
${\bar{\bar{R}}}_{xx}^{\prime }={\bar{\bar{U}}}_{x}\cdot {\bar{\bar{\Sigma }}}_{x}\cdot {\bar{\bar{V}}}_{x}^{†},\;$ ${\bar{\bar{R}}}_{zz}^{\prime }={\bar{\bar{U}}}_{z}\cdot {\bar{\bar{\Sigma }}}_{z}\cdot {\bar{\bar{V}}}_{z}^{†}$
Construct null spaces ${\bar{\bar{U}}}_{xn}$ and ${\bar{\bar{U}}}_{zn}$ out of ${\bar{\bar{R}}}_{xx}^{\prime }$ and ${\bar{\bar{R}}}_{zz}^{\prime }$, respectively, from the last $N_{f}-M$ columns of ${\bar{\bar{U}}}_{x}$ and ${\bar{\bar{U}}}_{z}$Construct matrices ${\bar{\bar{B}}}_{xG}=\left[{\bar{b}}_{x}\left(\phi_{1}\right),\cdots ,{\bar{b}}_{x}\left(\phi_{N_{g}}\right)\right]$ and ${\bar{\bar{B}}}_{zG}=\left[{\bar{b}}_{z}\left(\phi_{1}\right),\cdots ,{\bar{b}}_{z}\left(\phi_{N_{g}}\right)\right]$, where $\phi_{g}=-70^{{}^{\circ}}+\left(g-1\right)\Delta \phi_{g}$, $\Delta \phi_{g}=140^{{}^{\circ}}/\left(N_{g}-1\right)$ and
${\bar{b}}_{x}\left(\phi \right)=\left[1,e^{j2\pi f_{0}\tau_{x}\left(\phi \right)},\cdots ,e^{j2\pi f_{0}N_{f}\tau_{x}\left(\phi \right)}\right],\;$ ${\bar{b}}_{z}\left(\phi \right)=\left[1,e^{j2\pi f_{0}\tau_{z}\left(\phi \right)},\cdots ,e^{j2\pi f_{0}N_{f}\tau_{z}\left(\phi \right)}\right]$
Compute the DOA spectra
$P_{\mathrm{SSM},x}\left(\phi_{g}\right)={∥{\bar{\bar{U}}}_{xn}^{†}\cdot {\bar{b}}_{x}\left(\phi_{g}\right)∥}^{-2},\;1\leq g\leq N_{g},\;$ $P_{\mathrm{SSM},z}\left(\phi_{g}\right)={∥{\bar{\bar{U}}}_{zn}^{†}\cdot {\bar{b}}_{z}\left(\phi_{g}\right)∥}^{-2},\;1\leq g\leq N_{g}$
Pick *M* highest peaks from each spectrum and record their associated angles in ${\bar{\phi }}_{1x}$ and ${\bar{\phi }}_{1z}$, respectively.For every entry in ${\bar{\phi }}_{1z}$, find a best match in ${\bar{\phi }}_{1x}$ with DOA difference smaller than 0.5°, then record the average of both DOAs in ${\bar{\theta }}_{1}^{\prime }$.


### 3.2. Joint-ESPRIT in Projected Subspace

The DOAs of the signal sources with known CF ($f_{0}$) are estimated and stored in ${\bar{\theta }}_{1}^{\prime }$. The steering vectors associated with these sources are then removed by applying the orthogonal complement projectors

(11) $$\begin{array}{c} {\bar{\bar{\Pi }}}_{x}={\bar{\bar{I}}}_{2N_{s}+1}-{\bar{\bar{B}}}_{x}^{\prime }\cdot {\left({\bar{\bar{B}}}_{x}^{\prime †}\cdot {\bar{\bar{B}}}_{x}^{\prime }\right)}^{-1}\cdot {\bar{\bar{B}}}_{x}^{\prime †}\\ {\bar{\bar{\Pi }}}_{z}={\bar{\bar{I}}}_{2N_{s}+1}-{\bar{\bar{B}}}_{z}^{\prime }\cdot {\left({\bar{\bar{B}}}_{z}^{\prime †}\cdot {\bar{\bar{B}}}_{z}^{\prime }\right)}^{-1}\cdot {\bar{\bar{B}}}_{z}^{\prime †} \end{array}$$

where $N_{s}$ is the largest lag in $\Gamma_{s}$ of the second-order virtual arrays, ${\bar{\bar{B}}}_{x}^{\prime }$ and ${\bar{\bar{B}}}_{z}^{\prime }$ are the steering matrices of CPAs in *x* and *z* directions, respectively, with contiguous lags specified in $\Gamma_{s}$.

Next, apply the joint-ESPRIT (JE) algorithm to estimate the DOA and CF of the remaining sources embedded in the residual signals [8]. Construct two subarrays out of each second-order virtual array in *x* and *z* direction, respectively, as

(12) $$\begin{array}{c} \;{\bar{y}}_{x1}^{\prime \prime }\left[q\right]={\bar{\bar{\Pi }}}_{x}\cdot {\bar{y}}_{x1}^{\Phi }\left[q\right]={\bar{\bar{\Pi }}}_{x}\cdot {\bar{\bar{B}}}_{x1}^{\Phi }\cdot \bar{P}\left[q\right]={\bar{\bar{B}}}_{x1}^{\prime \prime }\cdot \bar{P}\left[q\right]\\ \;{\bar{y}}_{x2}^{\prime \prime }\left[q\right]={\bar{\bar{\Pi }}}_{x}\cdot {\bar{y}}_{x2}^{\Phi }\left[q\right]={\bar{\bar{\Pi }}}_{x}\cdot {\bar{\bar{B}}}_{x2}^{\Phi }\cdot \bar{P}\left[q\right]={\bar{\bar{B}}}_{x2}^{\prime \prime }\cdot \bar{P}\left[q\right]\\ \;{\bar{y}}_{z1}^{\prime \prime }\left[q\right]={\bar{\bar{\Pi }}}_{z}\cdot {\bar{y}}_{z1}^{\Phi }\left[q\right]={\bar{\bar{\Pi }}}_{z}\cdot {\bar{\bar{B}}}_{z1}^{\Phi }\cdot \bar{P}\left[q\right]={\bar{\bar{B}}}_{z1}^{\prime \prime }\cdot \bar{P}\left[q\right]\\ \;{\bar{y}}_{z2}^{\prime \prime }\left[q\right]={\bar{\bar{\Pi }}}_{z}\cdot {\bar{y}}_{z2}^{\Phi }\left[q\right]={\bar{\bar{\Pi }}}_{z}\cdot {\bar{\bar{B}}}_{z2}^{\Phi }\cdot \bar{P}\left[q\right]={\bar{\bar{B}}}_{z2}^{\prime \prime }\cdot \bar{P}\left[q\right] \end{array}$$

where ${\bar{y}}_{x1}^{\Phi }\left[q\right]$ and ${\bar{y}}_{x2}^{\Phi }\left[q\right]$ are the observation vectors from the first $2N_{s}$ and the last $2N_{s}$ second-order virtual sensors, respectively, in *x* direction, at the *q*-th sampling instant; ${\bar{\bar{B}}}_{x1}^{\Phi }$ and ${\bar{\bar{B}}}_{x2}^{\Phi }$ are the first $2N_{s}$ rows and the last $2N_{s}$ rows, respectively, of ${\bar{\bar{B}}}_{x}^{\Phi }$; ${\bar{\bar{B}}}_{x1}^{\prime \prime }={\bar{\bar{\Pi }}}_{x}\cdot {\bar{\bar{B}}}_{x1}^{\Phi }$ and ${\bar{\bar{B}}}_{x2}^{\prime \prime }={\bar{\bar{\Pi }}}_{x}\cdot {\bar{\bar{B}}}_{x2}^{\Phi }$. The vectors ${\bar{y}}_{z1}^{\Phi }\left[q\right]$, ${\bar{y}}_{z2}^{\Phi }\left[q\right]$ and the matrices ${\bar{\bar{B}}}_{z1}^{\Phi }$, ${\bar{\bar{B}}}_{z2}^{\Phi }$, ${\bar{\bar{B}}}_{z1}^{\prime \prime }$, ${\bar{\bar{B}}}_{z2}^{\prime \prime }$ are defined in the same way as their counterparts in *x*-direction. The matrices ${\bar{\bar{B}}}_{x1}^{\prime \prime }$, ${\bar{\bar{B}}}_{x2}^{\prime \prime }$, ${\bar{\bar{B}}}_{z1}^{\prime \prime }$ and ${\bar{\bar{B}}}_{z2}^{\prime \prime }$ satisfy rotational invariance property (RIP), namely,

(13) $$\begin{array}{c} {\bar{\bar{B}}}_{x2}^{\prime \prime }={\bar{\bar{B}}}_{x1}^{\prime \prime }\cdot {\bar{\bar{\Psi }}}_{x},\;\;{\bar{\bar{B}}}_{z2}^{\prime \prime }={\bar{\bar{B}}}_{z1}^{\prime \prime }\cdot {\bar{\bar{\Psi }}}_{z} \end{array}$$

with

$$\begin{array}{c} \;{\bar{\bar{\Psi }}}_{x}=\mathrm{diag}\left\{e^{j2\pi f_{M-M_{c}^{\prime }+1}\tau_{x}\left(\theta_{M-M_{c}^{\prime }+1}\right)},\cdots ,e^{j2\pi f_{M}\tau_{x}\left(\theta_{M}\right)}\right\}\\ \;{\bar{\bar{\Psi }}}_{z}=\mathrm{diag}\left\{e^{j2\pi f_{M-M_{c}^{\prime }+1}\tau_{z}\left(\theta_{M-M_{c}^{\prime }+1}\right)},\cdots ,e^{j2\pi f_{M}\tau_{z}\left(\theta_{M}\right)}\right\} \end{array}$$

which is prerequisite to applying the JE algorithm.

Define covariance matrices

(14) $$\begin{array}{c} {\bar{\bar{R}}}_{1}=E\left\{{\bar{y}}_{x1}^{\prime \prime }\left[q\right]\;{\bar{y}}_{z1}^{\prime \prime †}\left[q\right]\right\}={\bar{\bar{B}}}_{x1}^{\prime \prime }\cdot \bar{\bar{D}}\cdot {\bar{\bar{B}}}_{z1}^{\prime \prime †}\\ {\bar{\bar{R}}}_{2}=E\left\{{\bar{y}}_{x2}^{\prime \prime }\left[q\right]\;{\bar{y}}_{z1}^{\prime \prime †}\left[q\right]\right\}={\bar{\bar{B}}}_{x1}^{\prime \prime }\cdot {\bar{\bar{\Psi }}}_{x}\cdot \bar{\bar{D}}\cdot {\bar{\bar{B}}}_{z1}^{\prime \prime †}\\ {\bar{\bar{R}}}_{3}=E\left\{{\bar{y}}_{x1}^{\prime \prime }\left[q\right]\;{\bar{y}}_{z2}^{\prime \prime †}\left[q\right]\right\}={\bar{\bar{B}}}_{x1}^{\prime \prime }\cdot \bar{\bar{D}}\cdot \left({\bar{\bar{\Psi }}}_{z}^{†}\cdot {\bar{\bar{B}}}_{z1}^{\prime \prime †}\right)\\ {\bar{\bar{R}}}_{4}=E\left\{{\bar{y}}_{x2}^{\prime \prime }\left[q\right]\;{\bar{y}}_{z2}^{\prime \prime †}\left[q\right]\right\}=\left({\bar{\bar{B}}}_{x1}^{\prime \prime }\cdot {\bar{\bar{\Psi }}}_{x}\right)\cdot \bar{\bar{D}}\cdot \left({\bar{\bar{\Psi }}}_{z}^{†}\cdot {\bar{\bar{B}}}_{z1}^{\prime \prime †}\right), \end{array}$$

which can be implemented as

(15) $$\begin{array}{c} \;{\bar{\bar{R}}}_{1}=\frac{1}{Q}\sum_{q=1}^{Q}{\bar{y}}_{x1}^{\prime \prime }\left[q\right]\;{\bar{y}}_{z1}^{\prime \prime †}\left[q\right],\;\;{\bar{\bar{R}}}_{2}=\frac{1}{Q}\sum_{q=1}^{Q}{\bar{y}}_{x2}^{\prime \prime }\left[q\right]\;{\bar{y}}_{z1}^{\prime \prime †}\left[q\right],\\ \;{\bar{\bar{R}}}_{3}=\frac{1}{Q}\sum_{q=1}^{Q}{\bar{y}}_{x1}^{\prime \prime }\left[q\right]\;{\bar{y}}_{z2}^{\prime \prime †}\left[q\right],\;\;{\bar{\bar{R}}}_{4}=\frac{1}{Q}\sum_{q=1}^{Q}{\bar{y}}_{x2}^{\prime \prime }\left[q\right]\;{\bar{y}}_{z2}^{\prime \prime †}\left[q\right] \end{array}$$

The four matrices in (14) are concatenated to form

(16) $$\begin{array}{c} {\bar{\bar{R}}}_{c}=\left[\begin{array}{c} {\bar{\bar{R}}}_{1}\\ {\bar{\bar{R}}}_{2}\\ {\bar{\bar{R}}}_{3}\\ {\bar{\bar{R}}}_{4} \end{array}\right]={\bar{\bar{B}}}_{c}\cdot \bar{\bar{D}}\cdot {\bar{\bar{B}}}_{z1}^{\prime \prime †},\;\;{\bar{\bar{B}}}_{c}=\left[\begin{array}{c} {\bar{\bar{B}}}_{x1}^{\prime \prime }\\ {\bar{\bar{B}}}_{x1}^{\prime \prime }\cdot {\bar{\bar{\Psi }}}_{x}\\ {\bar{\bar{B}}}_{x1}^{\prime \prime }\cdot {\bar{\bar{\Psi }}}_{z}^{†}\\ {\bar{\bar{B}}}_{x1}^{\prime \prime }\cdot {\bar{\bar{\Psi }}}_{x}\cdot {\bar{\bar{\Psi }}}_{z}^{†} \end{array}\right] \end{array}$$

Then, apply SVD on ${\bar{\bar{R}}}_{c}$ to have

$$\begin{array}{c} {\bar{\bar{R}}}_{c}=\left[{\bar{\bar{U}}}_{s}\;\;{\bar{\bar{U}}}_{n}\right]\left[\begin{array}{cc} {\bar{\bar{\Sigma }}}_{s} & 0\\ 0 & 0 \end{array}\right]\left[\begin{array}{c} {\bar{\bar{V}}}_{s}^{†}\\ {\bar{\bar{V}}}_{n}^{†} \end{array}\right], \end{array}$$

where

(17) $$\begin{array}{c} {\bar{\bar{U}}}_{s}={\left[\begin{array}{c} {\bar{\bar{U}}}_{s1}\\ {\bar{\bar{U}}}_{s2}\\ {\bar{\bar{U}}}_{s3}\\ {\bar{\bar{U}}}_{s4} \end{array}\right]}_{8N_{s}\times \left(M-M_{c}^{\prime }\right)}={\bar{\bar{B}}}_{c}\cdot \bar{\bar{T}} \end{array}$$

spans the same subspace as ${\bar{\bar{B}}}_{c}$ does, and $M_{c}^{\prime }$ is the number of signals with known CF, estimated in the first stage.

By substituting (16) into (17), we have

$$\begin{array}{c} \;{\bar{\bar{U}}}_{s1}={\bar{\bar{B}}}_{x1}^{\prime \prime }\cdot \bar{\bar{T}}\\ \;{\bar{\bar{U}}}_{s2}={\bar{\bar{B}}}_{x1}^{\prime \prime }\cdot {\bar{\bar{\Psi }}}_{x}\cdot \bar{\bar{T}}={\bar{\bar{U}}}_{s1}\cdot {\bar{\bar{T}}}^{-1}\cdot {\bar{\bar{\Psi }}}_{x}\cdot \bar{\bar{T}}\\ \;{\bar{\bar{U}}}_{s3}={\bar{\bar{B}}}_{x1}^{\prime \prime }\cdot {\bar{\bar{\Psi }}}_{z}^{†}\cdot \bar{\bar{T}}={\bar{\bar{U}}}_{s1}\cdot {\bar{\bar{T}}}^{-1}\cdot {\bar{\bar{\Psi }}}_{z}^{†}\cdot \bar{\bar{T}}\\ \;{\bar{\bar{U}}}_{s4}={\bar{\bar{B}}}_{x1}^{\prime \prime }\cdot {\bar{\bar{\Psi }}}_{x}\cdot {\bar{\bar{\Psi }}}_{z}^{†}\cdot \bar{\bar{T}}={\bar{\bar{U}}}_{s1}\cdot {\bar{\bar{T}}}^{-1}\cdot {\bar{\bar{\Psi }}}_{x}\cdot {\bar{\bar{\Psi }}}_{z}^{†}\cdot \bar{\bar{T}} \end{array}$$

Next, take the inverse of ${\bar{\bar{U}}}_{s1}$ and multiply it to ${\bar{\bar{U}}}_{s2}$, ${\bar{\bar{U}}}_{s3}$ and ${\bar{\bar{U}}}_{s4}$, respectively, to derive

(18) $$\begin{array}{c} {\bar{\bar{U}}}_{12}={\bar{\bar{U}}}_{s1}^{-1}\cdot {\bar{\bar{U}}}_{s2}={\bar{\bar{T}}}^{-1}\cdot {\bar{\bar{\Psi }}}_{x}\cdot \bar{\bar{T}}\\ {\bar{\bar{U}}}_{13}={\bar{\bar{U}}}_{s1}^{-1}\cdot {\bar{\bar{U}}}_{s3}={\bar{\bar{T}}}^{-1}\cdot {\bar{\bar{\Psi }}}_{z}^{†}\cdot \bar{\bar{T}}\\ {\bar{\bar{U}}}_{14}={\bar{\bar{U}}}_{s1}^{-1}\cdot {\bar{\bar{U}}}_{s4}={\bar{\bar{T}}}^{-1}\cdot {\bar{\bar{\Psi }}}_{x}\cdot {\bar{\bar{\Psi }}}_{z}^{†}\cdot \bar{\bar{T}} \end{array}$$

By applying eigenvalue decomposition (EVD) on the average of these three matrices, we obtain

$$\begin{array}{c} \bar{\bar{\Omega }}=\frac{1}{3}\left({\bar{\bar{U}}}_{12}+{\bar{\bar{U}}}_{13}+{\bar{\bar{U}}}_{14}\right)={\bar{\bar{T}}}^{-1}\cdot \bar{\bar{\Lambda }}\cdot \bar{\bar{T}} \end{array}$$

where $\bar{\bar{\Lambda }}$ is a diagonal matrix containing all the eigenvalues of $\bar{\bar{\Omega }}$. The first two equations in (19) imply that

(19) $$\begin{array}{c} {\bar{\bar{\Psi }}}_{x}=\bar{\bar{T}}\cdot {\bar{\bar{U}}}_{12}\cdot {\bar{\bar{T}}}^{-1},\;\;{\bar{\bar{\Psi }}}_{z}={\left(\bar{\bar{T}}\cdot {\bar{\bar{U}}}_{13}\cdot {\bar{\bar{T}}}^{-1}\right)}^{†} \end{array}$$

Finally, the DOAs and CFs are estimated as

(20) $$\begin{array}{c} {\tilde{\theta }}_{m}=\tan^{-1}\frac{∠\Psi_{z,m}}{∠\Psi_{x,m}},\\ {\tilde{f}}_{m}=\frac{c}{2\pi d}\sqrt{{\left(∠\Psi_{x,m}\right)}^{2}+{\left(∠\Psi_{z,m}\right)}^{2}},\\ \;m=M_{c}^{\prime }+1,M_{c}^{\prime }+2,\cdots ,M \end{array}$$

where $\Psi_{x,m}$ and $\Psi_{z,m}$ are the ($m-M_{c}^{\prime }$)-th diagonal entries of ${\bar{\bar{\Psi }}}_{x}$ and ${\bar{\bar{\Psi }}}_{z}$, respectively. The proposed projected Joint-ESPRIT (PJE) algorithm is listed in Algorithm 2.

**Algorithm 2** Projected Joint-ESPRIT (PJE)
**Input:**
*Q* snapshots of manifold vectors, ${\bar{y}}_{x}^{\Phi }$ and ${\bar{y}}_{z}^{\Phi }$, in (7)Total number of signal sources, *M*Known carrier frequency, $f_{0}$Estimated DOA vector (${\bar{\theta }}_{1}^{\prime }$) from Algorithm 1
**Output:**
Estimated DOA vector, ${\bar{\theta }}^{\prime }$Estimated CF vector, ${\bar{f}}^{\prime }$
**Algorithm:**
Generate orthogonal complement projector ${\bar{\bar{\Pi }}}_{x}$ and ${\bar{\bar{\Pi }}}_{z}$ as in (11):
${\bar{\bar{\Pi }}}_{x}={\bar{\bar{I}}}_{2N_{s}+1}-{\bar{\bar{B}}}_{x}^{\prime }\cdot {\left({\bar{\bar{B}}}_{x}^{\prime †}\cdot {\bar{\bar{B}}}_{x}^{\prime }\right)}^{-1}\cdot {\bar{\bar{B}}}_{x}^{\prime †},$ ${\bar{\bar{\Pi }}}_{z}={\bar{\bar{I}}}_{2N_{s}+1}-{\bar{\bar{B}}}_{z}^{\prime }\cdot {\left({\bar{\bar{B}}}_{z}^{\prime †}\cdot {\bar{\bar{B}}}_{z}^{\prime }\right)}^{-1}\cdot {\bar{\bar{B}}}_{z}^{\prime †}$
Define ${\bar{y}}_{x1}^{\Phi }$ and ${\bar{y}}_{x2}^{\Phi }$ as the first $2N_{s}$ elements and last $2N_{s}$ elements, respectively, of ${\bar{y}}_{x}^{\Phi }$; ${\bar{y}}_{z1}^{\Phi }$ and ${\bar{y}}_{z2}^{\Phi }$ are the first $2N_{s}$ elements and last $2N_{s}$ elements, respectively, of ${\bar{y}}_{z}^{\Phi }$Retrieve residual signals as in (12):
${\bar{y}}_{x1}^{\prime \prime }={\bar{\bar{\Pi }}}_{x}\cdot {\bar{y}}_{x1}^{\Phi },$ ${\bar{y}}_{x2}^{\prime \prime }={\bar{\bar{\Pi }}}_{x}\cdot {\bar{y}}_{x2}^{\Phi },$ ${\bar{y}}_{z1}^{\prime \prime }={\bar{\bar{\Pi }}}_{z}\cdot {\bar{y}}_{z1}^{\Phi },$ ${\bar{y}}_{z2}^{\prime \prime }={\bar{\bar{\Pi }}}_{z}\cdot {\bar{y}}_{z2}^{\Phi }$
Estimate cross-covariance matrices as in (15):
${\bar{\bar{R}}}_{1}=\frac{1}{Q}\sum_{q=1}^{Q}{\bar{y}}_{x1}^{\prime \prime }\left[q\right]\;{\bar{y}}_{z1}^{\prime \prime †}\left[q\right],\;$ ${\bar{\bar{R}}}_{2}=\frac{1}{Q}\sum_{q=1}^{Q}{\bar{y}}_{x2}^{\prime \prime }\left[q\right]\;{\bar{y}}_{z1}^{\prime \prime †}\left[q\right],\;$ ${\bar{\bar{R}}}_{3}=\frac{1}{Q}\sum_{q=1}^{Q}{\bar{y}}_{x1}^{\prime \prime }\left[q\right]\;{\bar{y}}_{z2}^{\prime \prime †}\left[q\right],\;$

${\bar{\bar{R}}}_{4}=\frac{1}{Q}\sum_{q=1}^{Q}{\bar{y}}_{x2}^{\prime \prime }\left[q\right]\;{\bar{y}}_{z2}^{\prime \prime †}\left[q\right],$
Perform SVD to have $\bar{\bar{R}}={\left[{\bar{\bar{R}}}_{1},{\bar{\bar{R}}}_{2},{\bar{\bar{R}}}_{3},{\bar{\bar{R}}}_{4}\right]}^{t}=\bar{\bar{U}}\cdot \bar{\bar{\Sigma }}\cdot \bar{\bar{V}}$Define ${\bar{\bar{U}}}_{s}$ as the first $M-M_{c}^{\prime }$ columns of $\bar{\bar{U}}$, where $M_{c}^{\prime }$ is the size of ${\bar{\theta }}_{1}^{\prime }$Define ${\bar{\bar{U}}}_{s1}$ as the first $2N_{s}$ rows of ${\bar{\bar{U}}}_{s}$, ${\bar{\bar{U}}}_{s2}$ as the second $2N_{s}$ rows of ${\bar{\bar{U}}}_{s}$. ${\bar{\bar{U}}}_{s3}$ and ${\bar{\bar{U}}}_{s4}$ are defined similarly.Compute (19):
${\bar{\bar{U}}}_{12}={\bar{\bar{U}}}_{s1}^{-1}\cdot {\bar{\bar{U}}}_{s2},\;$ ${\bar{\bar{U}}}_{13}={\bar{\bar{U}}}_{s1}^{-1}\cdot {\bar{\bar{U}}}_{s3},\;$ ${\bar{\bar{U}}}_{14}={\bar{\bar{U}}}_{s1}^{-1}\cdot {\bar{\bar{U}}}_{s4}$
Perform eigenvalue decomposition (EVD) to have $\bar{\bar{\Omega }}=\left({\bar{\bar{U}}}_{12}+{\bar{\bar{U}}}_{13}+{\bar{\bar{U}}}_{14}\right)/3={\bar{\bar{T}}}^{-1}\cdot \bar{\bar{\Lambda }}\cdot \bar{\bar{T}}$Retrieve matrices ${\bar{\bar{\Psi }}}_{x}$ and ${\bar{\bar{\Psi }}}_{z}$ as in (19):
${\bar{\bar{\Psi }}}_{x}=\bar{\bar{T}}\cdot {\bar{\bar{U}}}_{12}\cdot {\bar{\bar{T}}}^{-1},\;$ ${\bar{\bar{\Psi }}}_{z}={\left(\bar{\bar{T}}\cdot {\bar{\bar{U}}}_{13}\cdot {\bar{\bar{T}}}^{-1}\right)}^{†}$
Compute remaining DOAs and CFs as in (21) to have ${\bar{\theta }}_{2}^{\prime }$ and ${\bar{f}}_{2}^{\prime }$.Record ${\bar{\theta }}^{\prime }={\left[{\bar{\theta }}_{1}^{\prime t},\;{\bar{\theta }}_{2}^{\prime t}\right]}^{t}$ and ${\bar{f}}^{\prime }={\left[f_{0},\;\cdots ,\;f_{0},\;{\bar{f}}_{2}^{\prime t}\right]}^{t}$


## 4. Simulations and Discussions

### 4.1. Simulation Setup

Two CPA(5, 6), each composed of 15 physical sensors, are deployed along *x* and *z* axes, respectively. The unit spacing is d=3 cm, which is λ/2 at 5 GHz. A total of M=30 sources are considered, including $M_{u}$ CF-unknown sources and $M_{c}$ CF-known sources. For convenience, we assume the first $M_{c}$ sources have known carrier frequency of $f_{0}$. Half of the DOAs are distributed over $\left[20^{{}^{\circ}},70^{{}^{\circ}}\right]$ and the other half over $\left[-70^{{}^{\circ}},-20^{{}^{\circ}}\right]$, all at uniform spacing.

Each source signal is assumed to display quasi-stationary property [11]. The time-frame length of each source signal is set to L=500. A total of 200 Monte Carlo realizations are simulated in each scenario. The signal-to-noise ratio (SNR) is defined as

(21) $$\begin{array}{c} \mathrm{SNR}=\frac{\frac{1}{LQ}\sum_{\ell =1}^{LQ}\left(E\left\{{∥{\bar{\bar{A}}}_{x}\cdot \bar{w}\left[\ell \right]∥}^{2}\right\}+E\left\{{∥{\bar{\bar{A}}}_{z}\cdot \bar{w}\left[\ell \right]∥}^{2}\right\}\right)}{E\left\{{∥{\bar{n}}_{x}\left[\ell \right]∥}^{2}\right\}+E\left\{{∥{\bar{n}}_{z}\left[\ell \right]∥}^{2}\right\}} \end{array}$$

The CF-known sources emit at $f_{0}=5$ GHz. The CFs of the remaining sources are chosen from $\left[0,f_{N}-B\right]$, with $f_{N}=6$ GHz and B=50 MHz, where the spacing between two adjacent channels is wider than *B*. To evaluate the performance of the proposed algorithm, define root-mean-square error (RMSE) as

$$\begin{array}{c} {\mathrm{RMSE}}_{\theta }=\frac{1}{180}\sqrt{\frac{1}{M}\sum_{m=1}^{M}{\left|{\tilde{\theta }}_{m}-\theta_{m}\right|}^{2}}\\ {\mathrm{RMSE}}_{f}=\frac{1}{f_{N}}\sqrt{\frac{1}{M_{u}}\sum_{m=M_{c}+1}^{M}{\left|{\tilde{f}}_{m}-f_{m}\right|}^{2}} \end{array}$$

where ${\tilde{\theta }}_{m}$ and $\theta_{m}$ are the estimated DOA and actual DOA, respectively, of source *m*, ${\tilde{f}}_{m}$ and $f_{m}$ are the estimated CF and actual CF, respectively, of the *m*-th CF-unknown source.

### 4.2. Cramer–Rao Bound

In [24,25], the Cramer–Rao bound (CRB) of DOA estimation was analyzed, assuming that there are fewer sources than antennas. In [26,27], the CRB of 1D DOA estimation in [24,25] was extended to 2D DOA estimation. In this work, the approach in [24,25] is extended to derive the CRB of joint DOA and frequency estimation. By concatenating $\bar{x}\left[\ell \right]$ and $\bar{z}\left[\ell \right]$ in (4), a signal vector is formed as

(22) $$\begin{array}{c} \bar{g}\left[\ell \right]=\left[\begin{array}{c} \bar{x}\left[\ell \right]\\ \bar{z}\left[\ell \right] \end{array}\right]=\left[\begin{array}{c} {\bar{\bar{A}}}_{x}\\ {\bar{\bar{A}}}_{z} \end{array}\right]\cdot \bar{w}\left[\ell \right]+\left[\begin{array}{c} {\bar{n}}_{x}\left[\ell \right]\\ {\bar{n}}_{z}\left[\ell \right] \end{array}\right]={\bar{\bar{A}}}_{g}\cdot \bar{w}\left[\ell \right]+{\bar{n}}_{g}\left[\ell \right] \end{array}$$

from which its covariance matrix is estimated as

(23) $$\begin{array}{c} {\bar{\bar{R}}}_{g}=\frac{1}{LQ}\sum_{\ell =1}^{LQ}\bar{g}\left[\ell \right]{\bar{g}}^{†}\left[\ell \right]=\frac{1}{Q}\sum_{q=1}^{Q}\frac{1}{L}\sum_{\ell =1}^{L}\bar{g}\left[\left(q-1\right)L+\ell \right]{\bar{g}}^{†}\left[\left(q-1\right)L+\ell \right]\\ =\frac{1}{Q}\sum_{q=1}^{Q}{\bar{\bar{A}}}_{g}\cdot {\bar{\bar{D}}}^{q}\cdot {\bar{\bar{A}}}_{g}^{†}+\sigma_{n}^{\prime 2}{\bar{\bar{I}}}_{2N}={\bar{\bar{A}}}_{g}\cdot {\bar{\bar{D}}}_{g}\cdot {\bar{\bar{A}}}_{g}^{†}+\sigma_{n}^{\prime 2}{\bar{\bar{I}}}_{2N} \end{array}$$

where ${\bar{\bar{D}}}_{g}=\frac{1}{Q}\sum_{q=1}^{Q}{\bar{\bar{D}}}^{q}=$ diag $\left\{\mu_{1},\cdots ,\mu_{M}\right\}$. The parameters are listed in a vector as

(24) $$\begin{array}{c} \bar{\eta }={\left[\theta_{1}^{\prime },\cdots ,\theta_{M}^{\prime },f_{0}^{\prime },f_{M_{c}+1}^{\prime },\cdots ,f_{M}^{\prime },\mu_{1},\cdots ,\mu_{M},\sigma_{n}^{\prime 2}\right]}^{t} \end{array}$$

where $\theta_{m}^{\prime }=\theta_{m}/180$ and $f_{m}^{\prime }=f_{m}/f_{N}$.

The CRB matrix is formulated as [24,25]

(25) B¯¯=σn′2LQReF¯¯†·Π¯¯g·F¯¯°D¯¯g′t−1

where $\sigma_{n}^{\prime 2}$ is the noise power defined in (4), LQ is the total number of snapshots, Re{} denotes the real part of its complex argument, ${\bar{\bar{\Pi }}}_{g}={\bar{\bar{I}}}_{2N}-{\bar{\bar{A}}}_{g}\cdot {\left({\bar{\bar{A}}}_{g}^{†}\cdot {\bar{\bar{A}}}_{g}\right)}^{-1}\cdot {\bar{\bar{A}}}_{g}^{†}$ is the orthogonal complement projection based on ${\bar{\bar{A}}}_{g}$, ∘ is the Hadamard product and ${\bar{\bar{D}}}_{g}^{\prime }=\left[\begin{array}{cc} {\bar{\bar{D}}}_{g} & {\bar{\bar{D}}}_{g}\\ {\bar{\bar{D}}}_{g} & {\bar{\bar{D}}}_{g} \end{array}\right]$, $\bar{\bar{F}}=\left[{\bar{\bar{A}}}_{\theta }^{\prime }\;,{\bar{\bar{A}}}_{f}^{\prime }\right]$, with

(26) $$\begin{array}{c} {\bar{\bar{A}}}_{\theta }^{\prime }=\left[\frac{\partial \bar{\alpha }\left(f_{0},\theta_{1}\right)}{\partial \theta_{1}},\cdots ,\frac{\partial \bar{\alpha }\left(f_{0},\theta_{M_{c}}\right)}{\partial \theta_{M_{c}}},\frac{\partial \bar{\alpha }\left(f_{M_{c}+1},\theta_{M_{c}+1}\right)}{\partial \theta_{M_{c}+1}},\cdots ,\frac{\partial \bar{\alpha }\left(f_{M},\theta_{M}\right)}{\partial \theta_{M}}\right]\\ {\bar{\bar{A}}}_{f}^{\prime }=\left[\frac{\partial \bar{\alpha }\left(f_{0},\theta_{1}\right)}{\partial f_{0}},\cdots ,\frac{\partial \bar{\alpha }\left(f_{0},\theta_{M_{c}}\right)}{\partial f_{0}},\frac{\partial \bar{\alpha }\left(f_{M_{c}+1},\theta_{M_{c}+1}\right)}{\partial f_{M_{c}+1}},\cdots ,\frac{\partial \bar{\alpha }\left(f_{M},\theta_{M}\right)}{\partial f_{M}}\right] \end{array}$$

where $\bar{\alpha }\left(f,\theta \right)={\left[{\bar{\alpha }}_{x}^{t}\left(f,\theta \right),\;{\bar{\alpha }}_{z}^{t}\left(f,\theta \right)\right]}^{t}$${\bar{\alpha }}_{x}\left(f,\theta \right)={\left[e^{j2\pi f\chi_{1}\tau_{x}\left(\theta \right)},\cdots ,e^{j2\pi f\chi_{N}\tau_{x}\left(\theta \right)}\right]}^{t}$, ${\bar{\alpha }}_{z}\left(f,\theta \right)={\left[e^{j2\pi f\chi_{1}\tau_{z}\left(\theta \right)},\cdots ,e^{j2\pi f\chi_{N}\tau_{z}\left(\theta \right)}\right]}^{t}$.

The SNR defined in (21) can be rewritten as

(27) $$\begin{array}{c} \mathrm{SNR}=\frac{\sum_{m=1}^{M_{c}}\mu_{m}{∥\bar{\alpha }\left(f_{0},\theta_{m}\right)∥}_{2}^{2}+\sum_{m=M_{c}+1}^{M}\mu_{m}{∥\bar{\alpha }\left(f_{m},\theta_{m}\right)∥}_{2}^{2}}{2\sigma_{n}^{\prime 2}} \end{array}$$

and the CRBs in DOA and CF estimations can be represented as

(28) $$\begin{array}{c} {\mathrm{CRB}}_{\theta }=\sqrt{\frac{1}{M}\sum_{m=1}^{M}B_{mm}}\\ {\mathrm{CRB}}_{f}=\sqrt{\frac{1}{M_{u}}\sum_{m=M+M_{c}+1}^{2M}B_{mm}} \end{array}$$

### 4.3. Maximum Detectable Number of Sources

**Figure 3:**
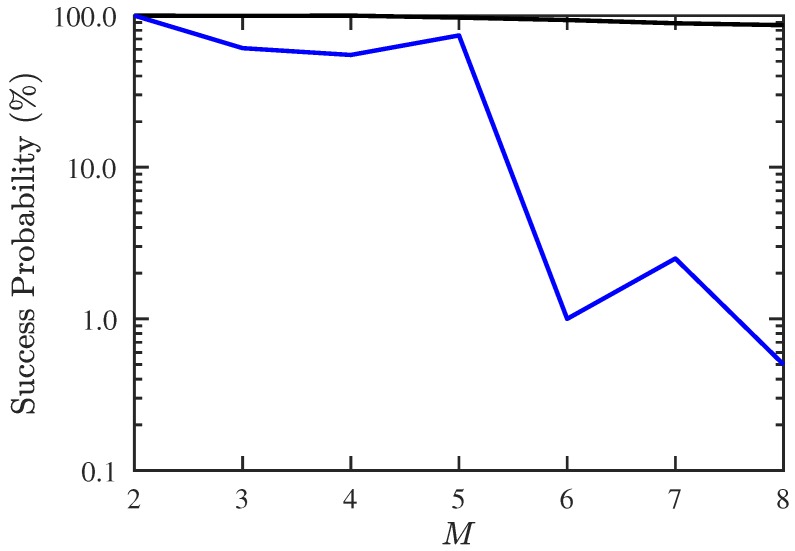
Success probability of DOA and CF estimation under different numbers of signals, SNR = 10 dB, Q=300, $M_{u}=1$. ───: proposed PJE algorithm, ───: conventional JE algorithm.

In the first scenario, we compare the maximum number of detectable signal sources when there is only one source with unknown CF (1.15 GHz). Figure 3 shows the success probability of DOA and CF estimation under different numbers of signal sources, where the success probability is defined as the number of Monte Carlo realizations with RMSE <5% in both DOA and CF estimation, divided by the total number of realizations. It is observed that the success probability with conventional JE [7,8] is acceptable when M≤5, and drops dramatically when M>5. On the other hand, the success probability with the proposed PJE remains about 90% even when M>5.

**Figure 4:**
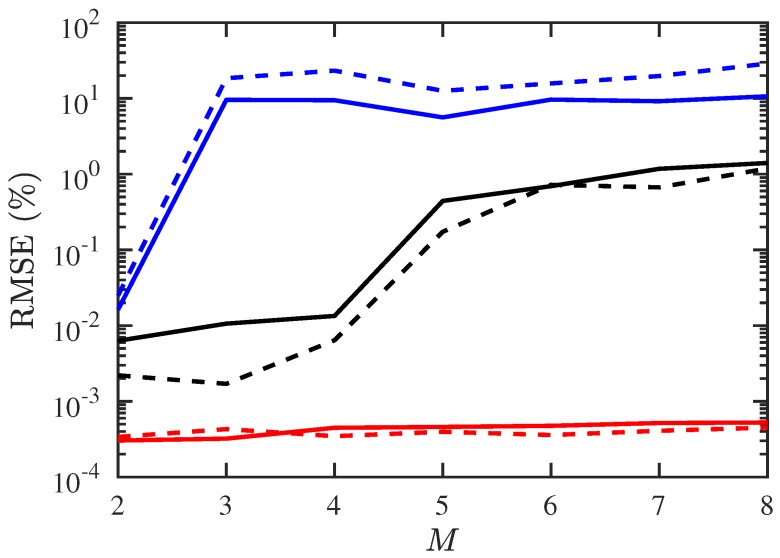
RMSE of DOA and CF estimation under different numbers of signals, SNR =10 dB, Q=300, $M_{u}=1$. ───: RMSE of DOA with proposed PJE algorithm, −−−: RMSE of CF with proposed PJE algorithm, ───: RMSE of DOA with conventional JE algorithm, −−−: RMSE of CF with conventional JE algorithm, ───: CRB of DOA estimation, −−−: CRB of CF estimation.

Figure 4 shows the RMSE of DOA and CF estimation, respectively, under different numbers of signal sources. The RMSE with conventional JE algorithm is higher than 5% when M>2, while that with the proposed PJE algorithm remains about 1% even at M=8.

**Figure 5:**
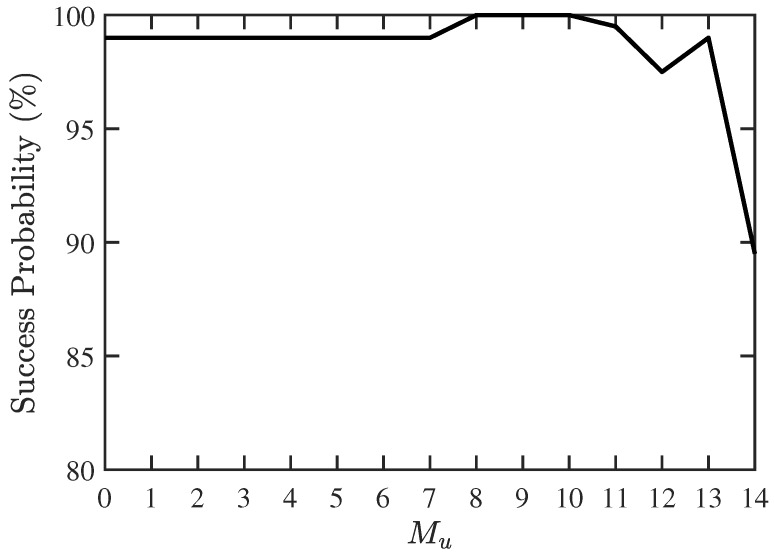
Success probability of DOA and CF estimation with proposed PJE algorithm, under different numbers of signals with unknown CF, SNR =10 dB, Q=300, M=30.

**Figure 6:**
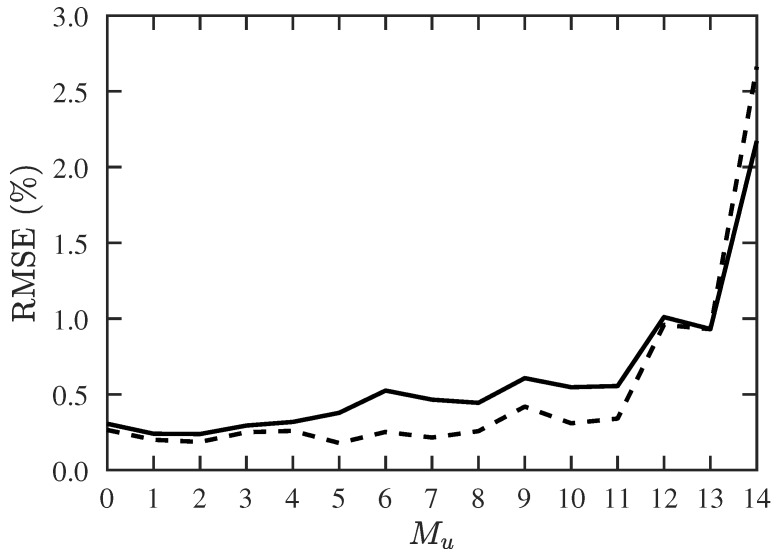
RMSE of DOA and CF estimation with proposed PJE algorithm, under different numbers of signals with unknown CF, SNR =10 dB, Q=300, M=30. ───: RMSE of DOA, −−−: RMSE of CF.

In the second scenario, we try to find the maximum detectable number of CF-unknown signal sources, under a fixed number of sources. The success probability and RMSE with the proposed PJE algorithm are shown in Figure 5 and Figure 6, respectively. The RMSE with the conventional JE algorithm is higher than 7%. The proposed two-stage algorithm works reasonably well when $M_{u}\leq 11$, and the RMSE increases dramatically when $M_{u}\geq 13$, indicating that the maximum number of CF-unknown sources is about 13 in this case.

**Figure 7:**
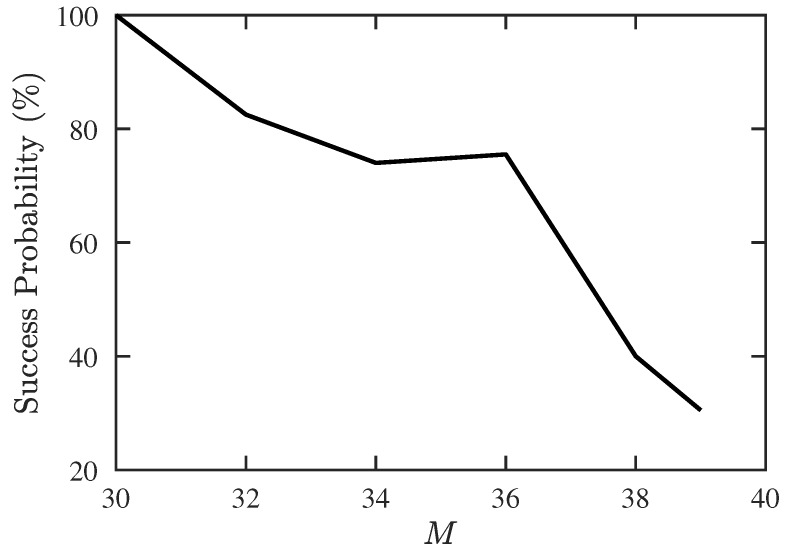
Success probability of DOA and CF estimation with proposed PJE algorithm, under different numbers of signal sources, SNR =10 dB, Q=300, $M_{u}=10$.

**Figure 8:**
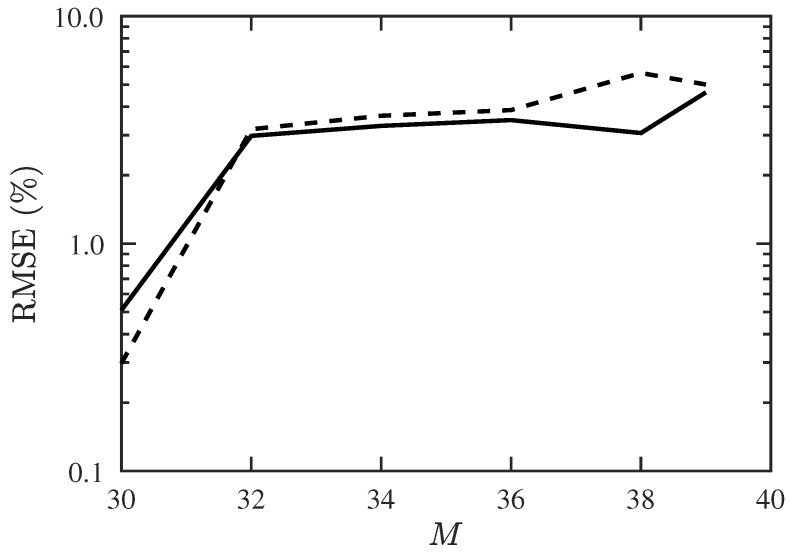
RMSE of DOA and CF estimation with proposed PJE algorithm, under different numbers of signal sources, SNR =10 dB, Q=300, $M_{u}=10$. ───: RMSE of DOA, −−−: RMSE of CF.

Next, we try to find the maximum allowable number of signal sources, including 10 CF-unknown sources. Figure 7 shows that the success probability decreases rapidly when M>36 and drops to 0 at M=40. Figure 8 shows that the RMSE of DOA and CF estimation increases significantly when *M* is increased from 30 to 32, and stays at the same level before *M* reaches 39.

### 4.4. Detail of Proposed Two-Stage Algorithm

**Figure 9:**
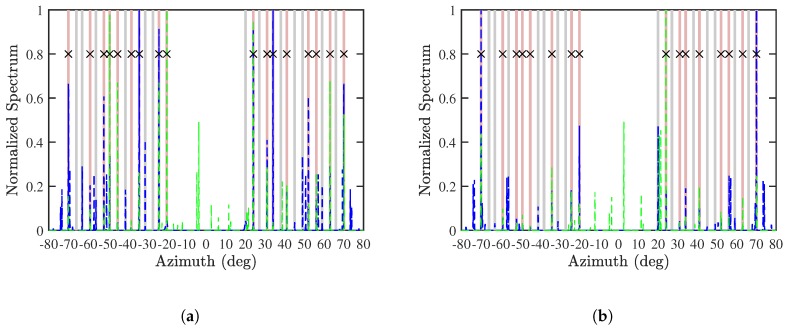
Normalized SS-MUSIC spectrum with Algorithm 1, (**a**) $M_{u}=13$ and (**b**) $M_{u}=14$, SNR = 10 dB, Q=300, M=30. ───: actual DOA of CF-known sources, ───: actual DOA of CF-unknown sources, −−−: estimated DOA with *x*-axis CPA, −−−: estimated DOA with *z*-axis CPA, ×: matched DOA.

Figure 9 shows the normalized SS-MUSIC spectrum with Algorithm 1. It is observed that the blue peaks are aligned with the green peaks at the actual DOAs of CF-known sources in both cases of $M_{u}=13$ and $M_{u}=14$. All the CF-known sources and some of the CF-unknown sources are accurately estimated.

**Figure 10:**
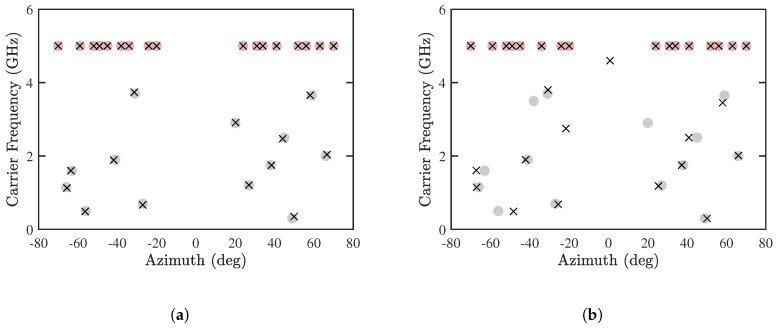
Joint estimation of DOA and CF with proposed PJE algorithm, (**a**) $M_{u}=13$ and (**b**) $M_{u}=14$, SNR = 10 dB, Q=300, M=30. •: CF-known sources, •: CF-unknown sources, ×: estimated sources.

Figure 10 shows the estimated DOA and CF, with $M_{u}=13$ and 14, respectively. In both cases, the DOA and CF of CF-known sources are accurately estimated. Figure 10a shows that all the CF-unknown sources are accurately estimated, while Figure 10b shows that some CF-unknown sources are poorly estimated, which is consistent with the observation in Figure 6 that the maximum number of detectable CF-unknown sources is 13.

**Figure 11:**
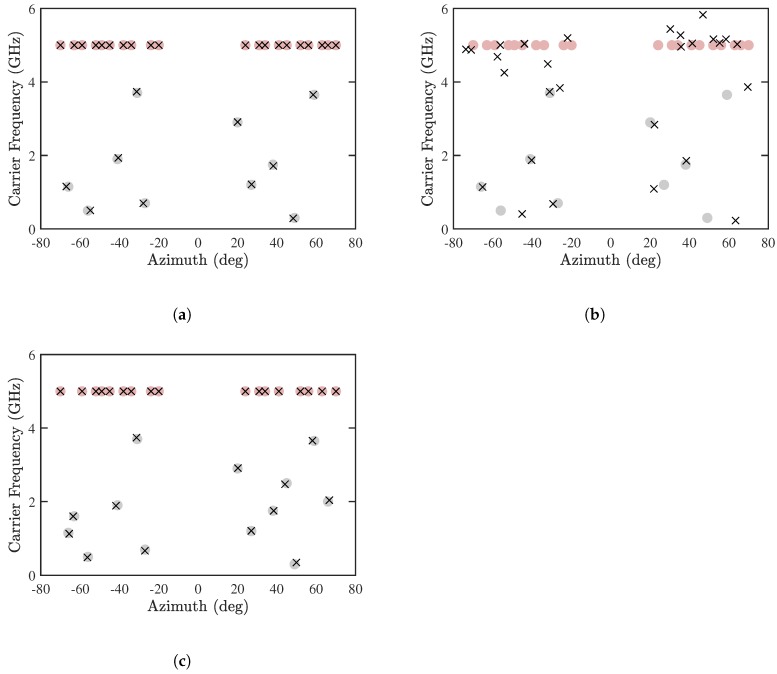
Joint estimation of DOA and CF, SNR = 10 dB, Q=300, M=30. (**a**) Proposed PJE algorithm with $M_{u}=10$, (**b**) conventional JE algorithm with $M_{u}=10$, (**c**) proposed PJE algorithm with $M_{u}=13$. •: CF-known sources, •: CF-unknown sources, ×: estimated sources.

Figure 11a,b show the estimated DOA and CF by using the proposed PJE algorithm and the conventional JE algorithm [8], respectively, with $M_{u}=10$ and M=30. It is observed that the proposed PJE algorithm can detect all the sources accurately, while the RMSE of either DOA or CF with the conventional JE algorithm exceeds 20%. It is interesting to observe in Figure 11b that the error of CF-known sources is higher than that of CF-unknown sources. Since the received signals are contributed by several CF-known sources directionally nearby the CF-unknown sources, a high resolution of signal phase is required to resolve these sources. The joint diagonalization in the conventional JE algorithm probably degrade such phase resolution. Figure 11c shows the results by using the proposed PJE algorithm with $M_{u}=13$. All the sources are accurately estimated, with slightly larger error than in the case with $M_{u}=10$.

The SS-MUSIC algorithm in [17] has been extended to deal with multiple sources, some with known CF and others with unknown CF. By matching the results from *x*-CPA and *z*-CPA, the DOAs of CF-known sources are estimated first. Then, the CF-known signals are removed from the received signals by using (11)–(13) in the proposed PJE algorithm. Finally, the conventional JE algorithm is applied to jointly estimate the DOA and CF of the remaining CF-unknown signals.

### 4.5. Robustness of Proposed Two-Stage Algorithm

**Figure 12:**
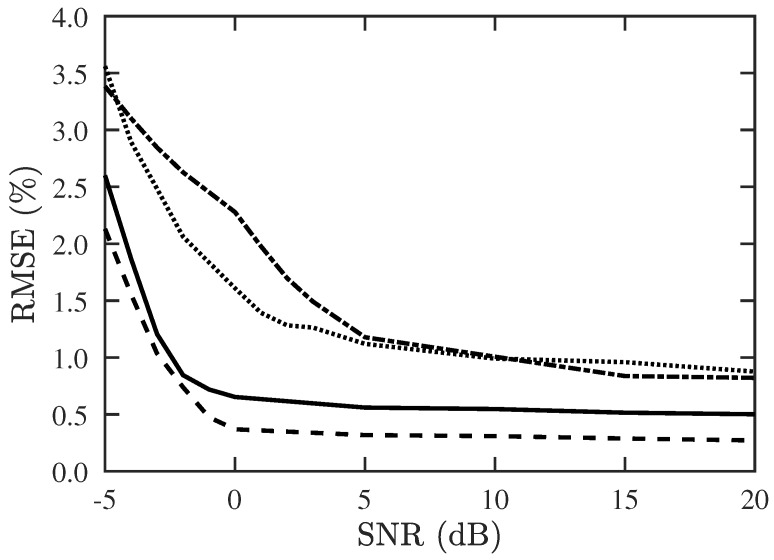
RMSE of DOA and CF estimation versus SNR, with proposed PJE algorithm, Q=300, M=30. ───: RMSE of DOA with $M_{u}=10$, −−−: RMSE of CF with $M_{u}=10$, −·−: RMSE of DOA with $M_{u}=13$, ⋯: RMSE of CF with $M_{u}=13$.

Figure 12 shows the RMSE of DOA and CF estimation with the proposed PJE algorithm, under different SNR. The RMSE is well below 1% at SNR >0 dB if $M_{u}=10$, and is below 1% at SNR >5 dB if $M_{u}=13$.

**Figure 13:**
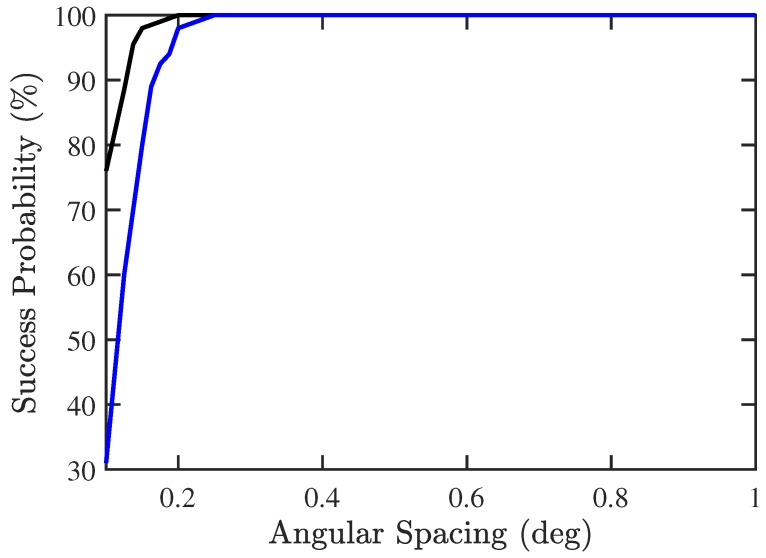
Success probability of DOA and CF estimation versus angular spacing, SNR =10 dB, Q=300, M=3, $M_{u}=1$. ───: proposed PJE algorithm, ───: conventional JE algorithm.

In the third scenario, we try to find the minimum angular spacing between two signal sources that can be detected by using the proposed two-stage algorithm. Consider the case of M=3, with the incident angles of $-60^{{}^{\circ}}$, $5^{{}^{\circ}}$ and ${\left(5+x\right)}^{{}^{\circ}}$, respectively, where *x* is in the range of [0.1,1]. Figure 13 shows that the proposed PJE algorithm can detect two signals as close as $0.3^{{}^{\circ}}$ apart, with success probability higher than 95%, as compared to $0.5^{{}^{\circ}}$ if conventional JE algorithm is used.

**Figure 14:**
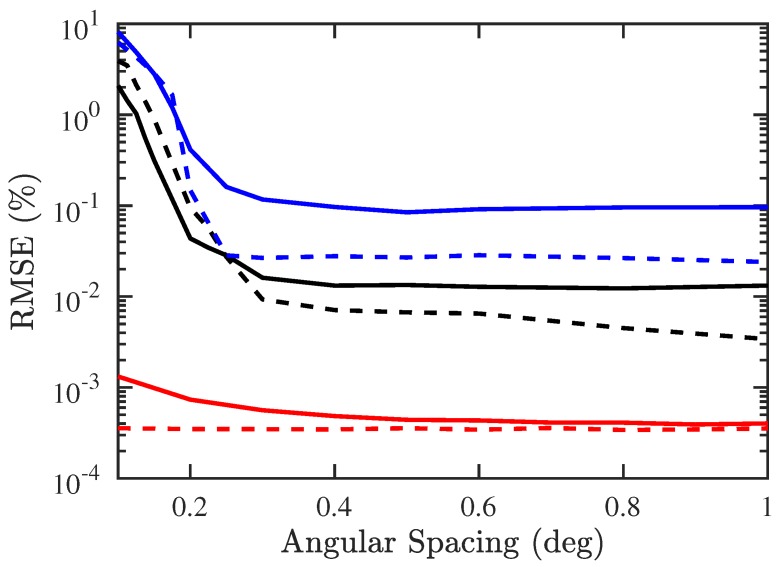
RMSE of DOA and CF estimation versus angular spacing, SNR =10 dB, Q=300, M=3, $M_{u}=1$. ───: RMSE of DOA with proposed PJE algorithm, −−−: RMSE of CF with proposed PJE algorithm, ───: RMSE of DOA with conventional JE algorithm, −−−: RMSE of CF with conventional JE algorithm, ───: CRB of DOA estimation, −−−: CRB of CF estimation.

Figure 14 shows that the RMSEs of both DOA and CF estimation by using the proposed PJE algorithm are smaller than their counterparts with conventional JE algorithm when the angular spacing is smaller than $0.5^{{}^{\circ}}$.

## 5. Conclusions

A two-stage method is proposed to estimate the DOA of multiple signal sources by processing the received signals contributed by multiple CF-known sources and multiple CF-unknown sources. In the first stage, the intermediate results derived from the received signals of two orthogonal CPAs are matched to estimate the DOA of CF-known sources. In the second stage, the proposed PJE algorithm is applied to the residual signals, after removing the contribution of CF-known sources, to estimate the DOA and CF of the remaining sources. Simulation results show that by applying the proposed two-stage method to received signals from two orthogonal CPA(5, 6), both DOA and CF of 30 signal sources, 13 of which with unknown CF, can be accurately estimated.
